# Perceived Barriers to Uptake of Family Planning Services Among Women of Reproductive Age in Osun State, Nigeria: Findings From a Qualitative Study

**DOI:** 10.7759/cureus.82823

**Published:** 2025-04-23

**Authors:** Adeniyi Fasanu, Oluwafunmilayo A Fasanu, Sunday C Adeyemo, Kehinde Awodele, Eniola D Olabode, Adegboye I Isawumi, Adeoye Oyewole, Lanre Olaitan

**Affiliations:** 1 Obstetrics and Gynecology, Osun State University, Osogbo, NGA; 2 Public Health, Adeleke University, Ede, NGA; 3 Health and Biomedical Sciences, Institut Superieur de Sante, Niamey, NER; 4 Psychiatry Medicine, Ladoke Akintola University of Technology, Ogbomosho, NGA; 5 Health Promotion and Environmental Health Education, University of Ilorin, Ilorin, NGA

**Keywords:** barrier, contraceptive methods, family planning, socio- cultural factors, spousal disapproval

## Abstract

Background

The Nigerian government has implemented several policies and programs aimed at improving access to and uptake of family planning services. However, despite these efforts, the country continues to face significant challenges, with a substantial proportion of women experiencing unmet needs for contraception and high rates of unintended pregnancies. Therefore, this study aims to assess the perceived barriers to the uptake of family planning services among women of reproductive age in Osun State, Nigeria.

Methods

The study employed a qualitative study design using focus group discussions (FGDs). Women of reproductive age, 15-49 years old, who are not using contraceptives and residing in the selected local government areas within Osun State were included in the study, while women who are not within reproductive age and women using contraceptives were excluded from the study. Participants were selected using a multi-stage sampling technique. One focus group discussion (one group consisting of five participants) was done in each market, giving a total of 12 focus group discussions with 60 participants. A key in-depth interview was also carried out with the permanent secretary of the Osun State Ministry of Health and the Family Planning program coordinator for Osun State. Data was collected using open-ended questions and conducted by doctors and public health professionals. The data was coded and analyzed using ATLAS.ti (Version 24) qualitative data analysis software. First, the interviews were transcribed and organized into themes and sub-themes based on the centrality of ideas within each category.

Results

Findings of the study highlighted four major barriers, which are: health concerns, spousal disapproval, religious and socio-cultural barriers such as misconceptions and stigma, and system barriers such as shortage of personnel as well as consumables.

Conclusion

Four primary factors in the evaluation of barriers and challenges to the uptake of family planning services in the study area include personal, spouse, religious and sociocultural, and system factors. Health education should provide accurate information about contraceptive methods, addressing myths and misconceptions. Collaboration with religious leaders can help address faith-based concerns.

## Introduction

Family planning (FP) is a conscious choice to space out pregnancies or decide how many children to have. This is done using different types of contraceptives, including hormonal methods, barrier methods, intrauterine devices (IUDs), and natural methods [1]. Barrier methods, such as male and female condoms, block sperm from reaching the egg and also help prevent sexually transmitted infections. Intrauterine devices (IUDs), which can be either hormonal or copper-based, can also be placed inside the uterus and act as a long-term way to prevent fertilization [2]. Hormonal contraceptives, like birth control pills or implants, work by changing a woman's hormones to stop ovulation [1]. Other natural methods of contraception include avoiding sex during fertile days, the lactational amenorrhea method, which delays ovulation during breastfeeding, and the withdrawal method, where the man pulls out before ejaculation [2]. Family planning (FP) is a key strategy to improve women‘s reproductive health and reinforce women‘s autonomy through informed choices about their sexual and reproductive needs and modern contraceptive use is an important indicator of reproductive health and FP program success [2].

The Nigerian government has implemented several policies and programs aimed at improving access to and uptake of family planning services. The Nigeria Family Planning Blueprint (Scale-Up Plan 2020-2024) aimed to achieve a modern Contraceptive Prevalence Rate (mCPR) target of 27% by the year 2024, outlining strategies to increase contraceptive use and reduce the incidence of unintended pregnancies [3]. Unintended pregnancy poses a wide array of health risks for both the mother and the child such as malnutrition, illness, abuse, neglect, and even death. It can also reduce educational and employment opportunities and increase poverty, which can go on to affect many generations [4].

However, despite these efforts, the country continues to face significant challenges, with a substantial proportion of women experiencing unmet needs for contraception and high rates of unintended pregnancies [1]. An estimated 218 million women have an unmet need for modern contraceptives, leading to 121 million unintended pregnancies annually [4]. The burden is particularly heavy in low- and middle-income countries (LMICs), where 66% of unintended pregnancies occur [5].

Osun State, located in south-western Nigeria, reflects the broader reproductive health challenges in the country. The state experiences high fertility rates as a result of significant unmet needs for contraception and a considerable burden of unintended pregnancy. Socio-cultural norms, including religious beliefs, myths about contraceptives and gender norms further hinder the uptake of family planning services [6].

An in-depth understanding of the barriers to uptake of family planning services among women of reproductive age will provide valuable insights for collaboration with local communities, religious groups, and women's organizations, which can help break down cultural barriers and promote awareness about family planning options, leading to increased uptake of family planning services. Therefore, this study aims to assess the perceived barriers to the uptake of family planning services among women of reproductive age in Osun State, Nigeria.

## Materials and methods

The study was carried out in Osun State, located in southwest Nigeria. The study employed a qualitative study design using Focus Group Discussions (FGDs). The study population was women of reproductive age, 15-49 years old.

Inclusion criteria

Inclusion criteria include women of reproductive age, 15-49 years old, irrespective of their marital status residing in the selected local government areas within Osun State, women who had past history of ever being pregnant irrespective of the outcome of the pregnancy and women with or without current contraceptive use at the time of study.

Exclusion criteria

Exclusion criteria include women who were less than 15 or over 49 years old during the study period and women who were residing outside the selected local government areas within Osun State during the study period.

Participants were selected using a multi-stage sampling technique; at the first stage, two local government areas were randomly selected (balloting method) from each of the three senatorial districts in Osun State. At the second stage, two high-volume markets were selected from each of the local government areas using random selection (balloting method), making 12 markets. The selected markets include Odo-ori and Oja-oba markets in Iwo Local Government, Oja-oba and Ola markets in Ejigbo Local Government, Ode-omu and Olufi markets in Ayedaade Local Government, Igbona market and Anoye market in Olorunda Local Government, Oke-ila and Ora markets in Ifedayo Local Government, and Iragbiji and Iree markets in Boripe Local Government. At the third stage, participants were selected by purposive sampling method from each market after the market women had been screened for eligibility criteria.

One focus group discussion (one group consisting of five participants) was done in each market, giving a total of 12 focus group discussions with 60 participants. A key in-depth interview was also carried out with the permanent secretary of the Osun State Ministry of Health and the Family Planning program coordinator for Osun State. Data was collected by doctors and public health professionals from 8th to 29th of April 2024. Separate interview guides were carefully designed for Focus Group Discussions and Key Informants to delve into the themes related to the socio-ecological model, health belief model, diffusion of innovation theory, and feminist theory, which together formed the conceptual framework of the study. The primary goal was to create a comprehensive guide that would facilitate in-depth discussions. To achieve this, open-ended questions were designed to elicit detailed responses while being mindful of ethical considerations. It was ensured that the questions were sensitive to the participants' privacy, consent, and overall well-being and respected the ethical principles of research, including confidentiality and voluntary participation. Interviews were primarily conducted in the local language or the language preferred by the respondents. All qualitative data were recorded, and notes were taken. At the end of each field day, the data were backed up on the study computer to minimize the risk of data loss.

Ethical approval for the study was obtained from the Adeleke University Review Ethical Committee and Osun State Health Research Ethics Committee (OSHREC) in the Ministry of Health. The study ensured that the sensitive data records were meticulously anonymized using unique identifiers, such as pseudo names, to safeguard the privacy and confidentiality of the individuals associated with the information.

All discussions from key informant interviews and focus group discussions were recorded. Thematic content analysis was adopted in the analysis of the data based on grounded theory. The interviews were transcribed, and then the transcripts were read and reread for emerging themes. These themes were later re-organised into themes and subthemes based on the centrality of ideas within each category. The data was coded and analyzed using ATLAS.ti (Version 24) qualitative data analysis software.

## Results

A total of 62 participants were recruited, 60 were involved in a focused group discussion, while two were involved in a key in-depth interview (Table 1).

**Table 1:** Sociodemographic Characteristics of Participants

Sociodemographic Characteristics (N=62)	Frequency	Percentage
Age (in years)		
15-19	10	16.1
20-24	18	29.0
25-29	12	19.3
30-34	8	12.9
35-39	5	8.1
40-44	5	8.1
45-49	4	6.5
Level of education		
Primary/Arabic	22	35.5
Secondary	28	45.2
Tertiary	12	19.4
Religion		
Christianity	32	51.6
Islam	30	48.4

Barriers to the uptake of family planning services

Four major themes emerged from the discussion on evaluating barriers and challenges to the uptake of family planning services in the study area. They include health concerns, spousal disapproval, system factors and religious and socio-cultural barriers. Under the system factors, four sub-themes emerged, which include: shortage of personnel, unavailability of family planning materials, difficulty reaching some areas and family planning staff requesting for money for a free service. Under the religious and socio-cultural barriers, stigmatization as a result of spotting, promiscuity and rivalry in a polygamous family emerged as sub-themes.

Health concerns

Among the issues that the women raised against family planning services included the concerns about how it could have different side effects on their body, such as irregular menstrual flows and changes in the body functionality (Table 2).

**Table 2:** Barriers to uptake of family planning services FGD: Focus Group Discussion; KII: Key in-depth interview

Themes	Excerpts from respondents’ responses
Health concerns	“I heard of a story my friend gave to me, she was like, when she did family planning after her second baby, she said it causes a woman’s body to change so she had to stop…” (25-29 years, Odo-ori, FGD)
	“I also heard that this family planning makes someone gets fat unnecessary and that it also affect menstrual cycle also” (15 – 24 years, Igbona, FGD)
	“Okay, for me, me, I do use the emergency, contraceptive pill like that. They say for three days. Yeah. So, me, I do use it. And I think, I don't want to use the pills again. So, it won't damage, my womb, or, damage anything in me. So, for me, I was thinking of going for that, that insertion, something. But someone told me that, no, someone that hasn’t given birth yet can’t use it because if you want to give birth it will be a problem and me i am not yet ready. So, I think, I don't want to use the pills again.” (15 – 24 years, Oke-Ila, FGD)
	“Another thing is you know our women you know they think the side effects do put some people off too, because when you talk about family planning methods It is part of the training that you tell your clients that there is something we call, bleeding changes or menstrual changes, it is just part of family planning. If a woman should take up the method menstrual changes is very much likely and so you need to tell from the onset that “ madam once you take up this method is very possible for you to have Menstrual changes, the menstrual flow might be more is might reduce, there might be spotting, there might be even amenorrhea” and once the client knows, automatically is to not be new to her when she experiencing such and so it is very, very you know those are the things…” (State Family Planning Programme Staff, KII)
	Family planning is ok but I have a lot of experience on family planning. My sister did family planning before for like every two months and she sees her period every day. So since that time, I do not think about family planning contraceptives…” (25-29 years, Anoye, FGD)
	“I do not support it. From the question being asked, some people are of the notion that a woman who submit herself for family planning would be falling sick from time to time due to the fact that the family planning has prevent some children from being born, that the children are hidden in the woman and in some case, the children may be chasing the woman even in her dream. That is why so women refused to opt for family planning….” (35-39 years, Iree, FGD)
	“Someone brought a 16 years child before to do family planning, does it have a side effect? Because I think someone that hasn't given birth, and the rumour I do hear, that I see is that it has side effects when you haven't given birth, right? That the time you are ready to give birth, that you start having issues. That it will cause infertility.” (15 – 24 years, Odeomu, FGD)
Spousal factors	“The fear that if a woman does not submit herself to her husband, he could flirt about….” (30-34 years, Olufi, FGD)
	“Some men detest family planning or for the wife to be taking drugs that will prevent her from becoming pregnant…” (25-29 years, Oja Oba, FGD)
Religious and socio-cultural barrier	“…and this one has been because we have a lot of barriers, rumours and misconception about family planning,” (State Family Planning Programme Staff, KII).
	“As I said earlier, ignorance is a disease. When people become aware, the complain would be minimal…” (25 – 29 years, Oke-Ila, FGD)
	“So a lot of things know misconception rumours that if you take up family planning, if you will not be able to give birth again, if you take up family planning, you will be becoming fat, emm bleeding might not stop, you know, by the time they start our IUD they are going to remove it from the head, you remove it from the leg, you know, you have a lot of rumors and misconception that affects low uptake of family planning, which is a major factor and what they don't know I do tell them this.” (State Family Planning Programme Staff, KII)
	“Let's say for me to go to a hospital to go and do family plan and they'll be looking at me as a wayward child and i am not a wayward child, i have my reasons, i have a very high libido when it comes to sex, i am sexually active…Yeah, that's me. So and I have to use something to protect myself. I don't want to get pregnant I'm not ready for a baby yet. So that's why I think those pills for me I now if I take it consistently that I don’t miss it, I won’t menstruate for like 3 months.” (24-29 years, Iragbiji, FGD)
	“…for example now for family planning so women cannot boldly say it outside that they are taking up family planning method, which should not be. Family planning is not a thing of shame and so looking at the community, the cultural aspects, if you are beside your sister in law, you might not be able to say it boldly, that you are uptake, the uptake of family planning is you know you are part of it…” (Ministry of Health Official, KII)
	“…especially when talking about the Muslim. The Muslim you know will say ahh I am unable to pray once i am having spotting you know once she spots she will not be able to pray and that is going to form a major barrier to the uptake. They say ahh once you take that method you will be spotty, and once you are spotty you will not be able to pray and I don't want anything to serve as a barrier to my prayer life so that one also serves as the major barrier to the uptake of family planning…” (30-34 years, Olufi, FGD)
	“People can say what they want but religious doctrines are the instructions of God. God said that we should not follow what people say but the general notion is that a woman who does family planning has the intention to engage in extra marital affairs but there is nothing that people would not say.” (20-24 years, Ora, FGD)
	“And again the perception of some people when they hear that a person is on family planning, they tend to look at the woman as a commercial sex worker and they belief that if you are on family planning, it is a certificate for you to be unfaithful to your husband, we can’t still take away that, some people still have such beliefs.” (Ministry of Health Official, KII)
	“Jealousy is another factor. Wives of a polygamous marriage could be jealous of one another. They may be competing as per number of children. It is also in the bible.” (40-44 years, Iragbiji, FGD)
	“Some churches encourage women to give birth to as many children as they can. My mother for instance, gave birth to twin at four different times. I am a twin. My mother has 11 surviving children. The first born has so many burdens to carry. May we not give birth to children we cannot take care of. We should embrace family planning.” (30-34 years, Olorunda, FGD)
	“you see I earlier mentioned that some religious organizations still don’t belief that we should even practise family planning in the first place.” (Ministry of Health Official, KII)
System factors	“But in terms of personnel, that is where we have challenge. This is not only applicable to family planning program but other programs too and this is not unconnected to the Japa syndrome, you train some people on family planning program; how they can help to administer these family planning methods to our clients and before you know it, there is alteration, some do retire and no replacement, some just decide to migrate to other health facilities such as federal government owned facilities and people out-rightly get out of the country for greener pastures, so those are the main challenges that we have concerning availability and quality family planning services.” (Ministry of Health Official, KII)
	“…, others in the aspect of the providers now, inadequate commodities and consumables, if a provider is at the facility, and the client is ready to take up method, and unfortunately, there is no commodities, there is no consumables to work with automatic that will form a kind of barrier to the uptake of that client….” State Family Planning Programme Staff, KII).
	“…So in terms of access to family planning, I still believe people have equal access. The only thing we cannot shy away from is that Osun state still has some hard to reach areas but I think with outreaches, they are trying to bridge those kind of gaps…” (Ministry of Health Official, KII)
	“And another challenge that we do face with is you know, we ask some client to and which can put them off once they come in automatically by the time they we have mobilizers, we have facility mobilizers once they talk to a client cleanse and say madam go to the facility, it is free. And here is the madam now, to take up coming to the facility and the provider now says, you have to pay a token to get the consumables they claim to be off, but I was told it was free, and so here now it is no more free, so the clients can decide to go away and thereby reducing the, the uptake. So we have a lot of challenges, especially in that aspect of inadequate consumables and commodities…” (State Family Planning Programme Staff, KII)
	“What I can say about that me, normally, not even going to the hospital for a family plan Just to go for a normal service I noticed the nurses are harsh and not friendly yeah, they should be friendly. It is an hospital where we take care of our health where we have a lot of issues I think that's where people work in daily even apart from banks, hospitals It is somewhere that people enter daily because we are too much in this earth I think our health care providers should be friendly very friendly, because me that I am sick I can’t get to the hospital and be expecting you to be harsh on me It will get the situation worse and not even talking of me going to do a family plan, I might be feeling low of myself before going there not me getting there and the doctor giving me a shallow reply Or making me feel like what I want to do is not right Yeah, it does make me feel outcast” (15 – 24 years, Igbona, FGD)

Spousal disapproval

Some of the women stated that they adopted the use of family planning services because they were concerned about their spouses being unable to control their sexual urges, and that inability to have sexual relations with their wives may make them promiscuous. However, some of these services were usually taken without the husband’s knowledge as they stated that it was possible for a man to not cooperate with his wife taking it. One of the women mentioned that a man was worried that family planning services could make his wife infertile (Table 2).

System factors

One of the primary system challenges is related to the shortage of personnel. The Ministry of Health staff expressed concern about how it has been hard to replace people who were retiring from work because of people relocating outside the country. Another issue of concern had to do with the availability of family planning materials, where the requisite materials were not sufficient to meet the needs of people. Also, there were remote communities that were often hard to reach because of their distance, and finally, there was the issue of family planning mobilisers who ask women to pay for accessing family planning materials, which are actually free, and thus put off the women from family planning. Women in the FGD discussions further talked about the unpleasant attitude of healthcare providers, which usually discourages them from seeking family planning services (Table 2).

Religious and socio-cultural barriers against family planning

Participants spoke about several norms and beliefs that could militate against women’s adoption of family planning services.

Most of these reasons were based on rumours, false beliefs and largely ignorance about family planning and how it worked. Also, some of the women also mentioned that family planning can lead to being stigmatized by others and that it could also counter their religious practices, such as when a Muslim woman is unable to observe her religious duties because of spotting. One of the most common ideologies was that sex education and subsequently adoption of family planning could promote promiscuity among women because they now know how to avoid getting pregnant while sleeping around or engaging in extra-marital affairs (Table 2).

Another cultural norm was that men have control over women’s bodies, and they could decide whether they should use contraception or not. Also, one of the women spoke about rivalry and jealousy in a polygamous home that may make women prioritise having children over uptaking family planning, irrespective of the existing number of children in the family. Other norms were associated with the role of religious institutions that encourage women to have unlimited children and also oppose family planning (Table 2).

## Discussion

Family planning is important for improving health and well-being, but many people face challenges in using it. These challenges can be personal, related to a spouse, influenced by society, or caused by problems in the healthcare system.

One of the most significant barriers identified was the fear of health complications associated with family planning methods. Many women expressed concerns about side effects such as irregular menstrual cycles, weight gain, prolonged bleeding, and potential damage to reproductive health. These fears were reinforced by personal experiences, other people’s experience and misinformation. Similarly, Adedini and Omisakin [6] reported similar concerns in their study among adolescents and young women. Some women believed that contraceptive methods, particularly hormonal ones, could lead to infertility, preventing them from conceiving in the future, as noted in recent studies, where women expressed concerns about their future ability to have children after using birth control [7]. Education and counseling can help people have a correct understanding of the benefits and potential side effects of family planning.

Spousal disapproval was also found to play an important role in contraceptive uptake. A husband’s opinion about family planning can affect a woman’s decision to use contraceptives. In some families, men make most of the decisions, and if they do not support birth control, their wives may not use it [8]. A recent study found that women who felt unsupported by their partners were more likely to avoid or discontinue contraceptive use [9]. In some cases, partners worry about potential side effects on their wives or see contraception as unnecessary if they prefer larger families [10]. Studies show that involving men in family planning education can help change their views and increase contraceptive use [11].

Cultural beliefs and societal norms were also identified as major barriers to family planning. Many misconceptions and rumors about contraceptives deterred women from using them. For instance, some respondents believed that family planning could lead to permanent infertility or cause prolonged bleeding. Others cited religious reasons, stating that spotting caused by contraceptives interfered with religious observances, particularly among Muslim women. Additionally, societal stigma surrounding family planning made some women reluctant to seek services, as they feared being labeled as promiscuous or irresponsible. Similarly, the study of Isa et al. [12] revealed similar concerns about violating cultural or religious beliefs. Biney et al. [13] also highlighted the role of societal acceptance in patriarchal settings. This highlights the need for culturally sensitive health education programs that work with community leaders and religious institutions to dispel myths and encourage informed decision-making.

Problems in the healthcare system can also make it hard for people to use family planning. Some clinics do not have enough contraceptive supplies, so women may not get the method they want. The cost of contraceptives is another issue, especially for people who cannot afford them. Some women also avoid family planning services because they feel healthcare workers judge them or do not respect their privacy [14]. Addressing these systemic issues requires targeted interventions such as strengthening healthcare workforce capacity, ensuring the availability of contraceptives, enforcing free service policies, and training providers on respectful and patient-centered care.

The strength of the study lies in the engagement of the Ministry of Health and Family Planning officials, which gave the first insight into the system factors affecting family planning. This can help to inform policymakers and other stakeholders. However, the study used a small sample size within six local governments in Osun State and therefore has limited generalizability of findings beyond Osun State.

## Conclusions

The findings of this study have important implications for public health programs and policies aimed at improving family planning uptake. The identified barriers - health concerns, spousal disapproval, system factors, and religious and socio-cultural influences - reveal the complexity of challenges that women face when trying to access contraceptive services. Health system issues such as shortages of trained personnel, unavailability of materials, and unofficial charges by healthcare workers contribute to poor service delivery and discourage use. These findings suggest the need for strengthening the healthcare system through better staffing, improved supply chains, and enhanced accountability. Furthermore, the presence of misinformation and fear of side effects highlights the need for targeted health education that addresses myths, promotes awareness, and encourages informed decision-making. Spousal disapproval and gender power imbalances were also notable barriers, indicating the importance of involving men in reproductive health discussions and promoting shared decision-making within households. Programs should also focus on empowering women to make autonomous choices about their reproductive health.

Religious and socio-cultural beliefs emerged as powerful influences, with stigmatization, fears of promoting promiscuity, and religious restrictions being commonly cited. This underscores the importance of engaging religious and community leaders in sensitization campaigns, as well as developing culturally respectful communication strategies. Addressing these deeply rooted norms will be essential for increasing acceptance and use of family planning services. This study serves as a baseline for future research which includes exploring the effectiveness of community-based interventions, strategies for male involvement, the role of cultural and religious leaders, and the impact of empowerment initiatives on women’s reproductive choices. Overall, the study provides a valuable foundation for designing more effective and inclusive family planning programs.
